# Mobile Phone Interventions for Sleep Disorders and Sleep Quality: Systematic Review

**DOI:** 10.2196/mhealth.7244

**Published:** 2017-09-07

**Authors:** Jong Cheol Shin, Julia Kim, Diana Grigsby-Toussaint

**Affiliations:** ^1^ Department of Kinesiology and Community Health University of Illinois-Urbana Champaign Champaign, IL United States; ^2^ Division of Nutritional Sciences University of Illinois-Urbana Champaign Urbana, IL United States

**Keywords:** mHealth, apps, mobile health, sleep

## Abstract

**Background:**

Although mobile health technologies have been developed for interventions to improve sleep disorders and sleep quality, evidence of their effectiveness remains limited.

**Objective:**

A systematic literature review was performed to determine the effectiveness of mobile technology interventions for improving sleep disorders and sleep quality.

**Methods:**

Four electronic databases (EBSCOhost, PubMed/Medline, Scopus, and Web of Science) were searched for articles on mobile technology and sleep interventions published between January 1983 and December 2016. Studies were eligible for inclusion if they met the following criteria: (1) written in English, (2) adequate details on study design, (3) focus on sleep intervention research, (4) sleep index measurement outcome provided, and (5) publication in peer-reviewed journals.

**Results:**

An initial sample of 2679 English-language papers were retrieved from five electronic databases. After screening and review, 16 eligible studies were evaluated to examine the impact of mobile phone interventions on sleep disorders and sleep quality. These included one case study, three pre-post studies, and 12 randomized controlled trials. The studies were categorized as (1) conventional mobile phone support and (2) utilizing mobile phone apps. Based on the results of sleep outcome measurements, 88% (14/16) studies showed that mobile phone interventions have the capability to attenuate sleep disorders and to enhance sleep quality, regardless of intervention type. In addition, mobile phone intervention methods (either alternatively or as an auxiliary) provide better sleep solutions in comparison with other recognized treatments (eg, cognitive behavioral therapy for insomnia).

**Conclusions:**

We found evidence to support the use of mobile phone interventions to address sleep disorders and to improve sleep quality. Our findings suggest that mobile phone technologies can be effective for future sleep intervention research.

## Introduction

Sleep disorders are an important public health problem that affects approximately 50 to 70 million people in the United States [1]. Sleep disorders are defined as having abnormal sleep behaviors, including insomnia, sleep apnea, restless leg syndrome, and narcolepsy [2]. Those who have chronic sleep disorders are at a greater risk for obesity, diabetes, hypertension, cardiovascular disease, stroke, and depression [3-6].

In addition to sleep disorders, insufficient sleep and irregular sleep patterns are also risk factors for obesity [7], impaired cardiovascular function [8,9], and diabetes [10]. According to the 2011-2014 report from the National Center for Health Statistics [11], 31.7% of US adults do not meet the National Sleep Foundation’s recommendation for at least 7 hours of sleep per night. Moreover, the 2011 Sleep in America poll reported that 63% of Americans did not meet the recommendations for sleep time during weekdays [12]. Given that sleep disorders and insufficient sleep increase the risk for chronic diseases, sleep interventions that improve sleep quality are increasingly becoming more important.

In 2011, approximately 39% of Americans, 72% of whom were adolescents, used mobile phones immediately before sleeping [12]. With the increasing use of mobile technology, mobile health (mHealth) is increasingly being used as a practical intervention tool in medicine and public health [13-19]. The term mHealth refers to the provision of health care services and delivery of personal health information using mobile technology such as mobile phones [20]. The portability of mobile devices used in mHealth addresses issues related to accessibility. This allows health care practitioners to provide services to patients regardless of geographical location [15,21]. Specifically, mHealth technology has additional unique functions that are not typically found in traditional care, such as text messaging-tailored medical advice or individualized phone alarms to encourage specific health behaviors (eg, exercising or taking medications) [22]. Regarding sleep, accelerometers in mobile phones can be used to measure and to evaluate sleep patterns [23], and voice recordings can detect abnormal sleep behavior such as snoring and sleep talking [24].

The potential and importance of mHealth technology in health care and health interventions are evident through more than 100,000 health apps in the app store, and a US $26 billion estimated mHealth market size in 2017 [25]. The 2016 mHealth App Developer Economics report [26] indicated that 53% of new apps were designed to improve or address various health conditions. In sum, mHealth is expected to play a significant role in health care due to its ability to be easily integrated into health care services and intervention studies.

### Research Objective

Although new mHealth technologies are assumed to be able to improve the quality and quantity of sleep, limited examinations of behavioral sleep interventions using mobile phones exist [13,22]. For example, previous studies [27-31] have focused on examining the technological aspects of mobile phones only (ie, calibration and validation studies, device feasibility). Moreover, previous studies examining the use of the mobile phone’s effectiveness for addressing sleep quality or quantity have only focused on one specific sleep disorder, such as sleep disturbance [32] or obstructive sleep apnea [33,34]. Therefore, the primary purpose of this review was to determine the effectiveness for sleep disorder improvement. We hypothesized that interventions with mobile technology have a positive impact on improving sleep quality and various sleep disorders (eg, sleep apnea, snoring, or insomnia).

## Methods

### Study Selection Criteria

This study included articles if they met the following criteria:

1. Study design: randomized controlled trials (RCTs), pre-post studies, and case-control studies;
2. Sleep intervention study: using mHealth technology (eg, a mobile device or app);
3. Intervention outcome: sleep outcome measurement (eg, Insomnia Severity Index [ISI], Pittsburgh Sleep Quality Index [PSQI], or Epworth Sleepiness Scale [ESS]);
4. Language: written in English; and
5. Article type: peer-reviewed publications.

### Search Strategy

Articles published between January 1983 (the year of the first handheld and commercial cellular phone from Motorola [35]) and December 2016 were searched from four electronic databases, including EBSCOhost: Academic Search Complete (ASC) & Cumulative Index to Nursing and Allied Health Literature (CINAHL), PubMed/Medline, Scopus, and Web of Science. The journal search process occurred from February 18 to 19, 2017. To identify components of three research topics, namely, “sleep,” “mHealth,” and “study design,” the specified search keywords were used as a search algorithm (see Multimedia Appendix 1).

After searching the electronic databases, one author (JCS) selected articles that were published in peer-reviewed journals and excluded books, case reports, conference proceedings, product reviews, newspapers, patents, serials, and theses. Secondly, the same author removed duplicate articles from the combined search results and screened articles based on the title and abstract. In addition, JCS hand-searched for relevant articles in the JMIR search engine on February 28, 2017. Once all full-text articles were found, two researchers (JCS and JK) reviewed each article using the eligibility criteria and eliminated unrelated articles. The entire procedure followed the guidelines for Preferred Reporting Items for Systematic Reviews and Meta-Analysis (PRISMA) [36].

### Data Extraction

The following information was extracted from each article included in the systematic review: first author’s name, publication year, the country where the study was conducted, sample size, sample age, sample characteristics, study period, retention rate, study design, the technology used in the intervention, and measurement of sleep outcome. The retention rate was calculated using the number of participants who completed all assessments for the intervention study divided by the number of individuals who were originally recruited. Intervention technology was identified using two approaches: (1) intervention using mobile phone app and (2) supplementary mobile phone usage with traditional intervention. Additional information was extracted by using the empirical approach of statistical analysis such as effect size, standard error, and standard deviation for the difference before and after interventions.

### Statistical Analysis

The effect size and standard deviation for each study’s sleep outcome measurement were obtained from data extraction. With each effect size, there was a difference of calculation for the total effect size based on the type of study. For example, the effect size of pre-post test was only considered the difference between pretest scores and posttest scores. On the other hand, the effect size of RCTs was calculated using the difference between the intervention and the control groups’ effect sizes. Based on the total effect size, sample size, and standard error, two-sample *t* tests were performed to determine the mobile phone intervention effectiveness.

### Study Quality Assessment

The study quality assessment tool was derived from Zhu and An [37], and then revised after discussion and peer review by the researchers for the purpose of this study. The quality of research was assessed using the following criteria: (1) the research question and objectives were stated clearly, (2) mobile health and/or mHealth was defined, (3) a control group was included, (4) participants were randomly recruited from a well-defined population, (5) sample size was more than 30, (6) attrition was analyzed and determined not to significantly differ by respondents’ baseline characteristics between control and experiment groups (<20%), (7) baseline characteristics between control and intervention groups were similar, (8) the intervention period was at least 4 weeks, (9) the sleep disorder measurement tools were shown to be reliable and valid in previous studies, and (10) demographic information was available to control for potential confounders in future analysis. A sample size of 30 was chosen due to a guarantee of a normal sample distribution based on the central limit theorem. The total study quality score was determined by summing items 1 to 10, and categories were created to define a general quality of research as poor (score 0-3); moderate (score 4-6), and high (score 7-10).

## Results

### Study Selection

**Figure 1:**
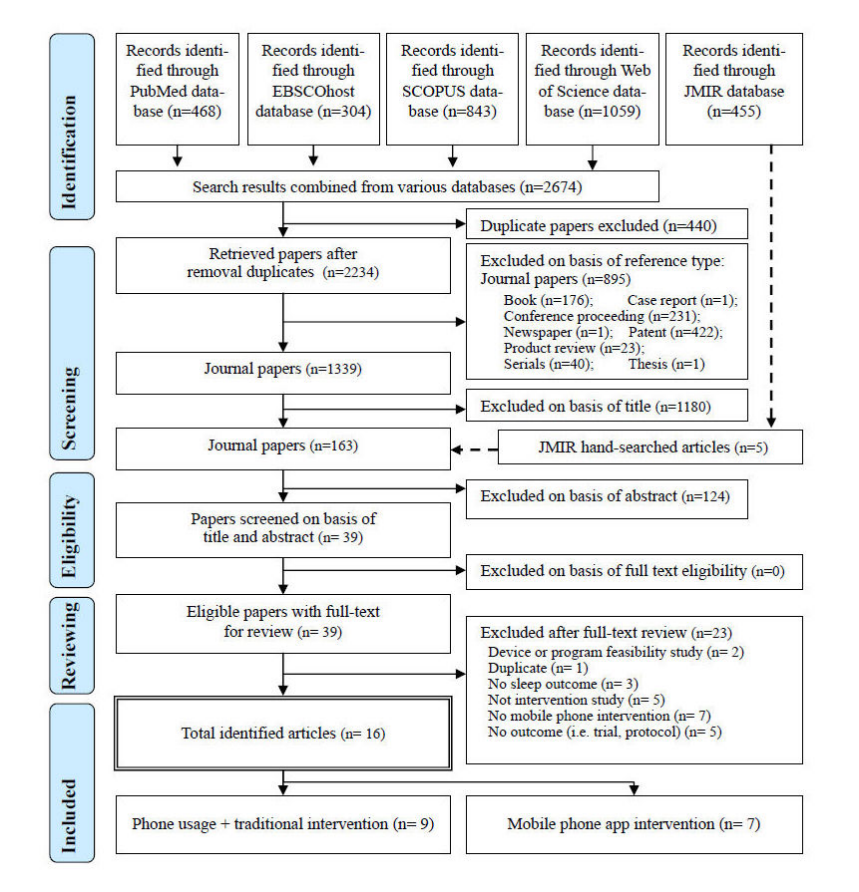
Study selection procedure according to the PRISMA guidelines.

The results of the literature search are summarized in Figure 1. A total of 2674 articles were initially selected from four electronic databases (EBSCOhost: CINAHL&ASC, PubMed/ Medline, Scopus, and Web of Science). After excluding 440 duplicate articles, 896 articles were excluded from the first screening of peer-reviewed journal articles and 1339 articles were left. After the first screening, five articles were included from hand searching, and 1304 were excluded based on the title (n=1180) and abstract (n=124). All 39 remaining articles were then downloaded as PDF files for full-text peer review and discussion [13-16,21,22,38-70]. We excluded 23 articles after the full-text screening for the following reasons: (1) a device or program feasibility study (n=2) [66,69], (2) duplicated article (n=1) [21], (3) not an intervention study (n=5) [39,43,47,49,53], (4) not a mobile phone intervention (n=7) [14,42,48,56,58,63,68], (5) no sleep outcome (n=3) [16,44,65], and (6) a protocol or trial (n=5) [40,45,50-52]. The entire selection procedure was independently performed by two researchers (JCS and JK), and then the research team resolved differences by discussion. From individual selections, the interrater reliability analysis using the kappa statistic was performed with peer reviewers to determine consistency among raters. The interrater reliability was found to be Cohen kappa=.80, representing substantial agreement [20]. Therefore, a total of 16 articles that satisfied the inclusion criteria were used for the final analysis.

### Study Characteristics

Table 1 provides summary information of individual studies. A total of 16 studies were included in the analysis: one case study [46], three pre-post studies [13,15,41], and 11 RCTs [21,22,38,54,55,57,59,61,62,64,67,70]. Of the 12 RCTs, three studies [62,64,67] used mobile phone apps as an intervention tool; one study [21] used text messages as part of the intervention. RCTs ranged in total sample size from 30 to 502 and had a larger sample size than pre-post studies, which ranged from three [13] to 12 [41]. The study period for RCTs was from 4 weeks to 6 months, whereas for pre-post studies it was between 2 and 5 weeks. For the study region, eight studies were performed in the Unites States [13,15,21,41,55,61,62,70], two studies in Canada [54,67], two studies in Asia (Hong Kong [57] and Taiwan [46]), and four studies in Europe (Finland [38], France [64], Netherlands [21], and Sweden [59]). Most of the studies were published after 2012, except one study that was published in 2006 [67]. Three studies [15,46,64] focused on an elderly population, whereas three studies [21,67,70] focused on a young adult population.

**Table 1:** Basic characteristics of the studies of mobile phone interventions on sleep disorders (N=16).

Author(s), Year	Study region	Sample characteristics^a^	Sample size	Mean age	Study period	Intervention methods^b^	Study design^c^	Sleep outcome measurement^d^
Anttalainen el al, 2014 [38]	Finland	OSA patients	111	55.27	3 m	Phone + CPAP	RCT	AHI, ESS
Babson et al, 2015 [13]	United States	Veterans with CUD	3	47	2 w	App: “CBT-I Coach” for iOS	Pre-post	PSQI
Bauer et al, 2012 [41]	United States	Metropolitan area adults	12	32	4 w	App: “ShutEye” for Android	Pre-post	ESS
Chen et al, 2015 [46]	Taiwan	Elderly female	1	64	5 w	App: “Win-Win A Sleep”	Case study	SSR
Filion et al, 2015 [21]	United States	Young adult smokers	116	18-25	6 w	Text message	RCT	SQ_PSQI, TST
Fox el al, 2012 [54]	Canada	OSA patients	75	53.54	3 m	Phone + CPAP	RCT	AHI, ESS
Freeman et al, 2015 [55]	United States	Breast cancer survivors	102	55.44	3 m	Phone-supported teleconference	RCT	PSQI
Ho et al, 2014 [57]	Hong Kong	Insomnia patients	149	38.5	12 w	Phone + CBT-I	RCT	PSQI, ISI, DBAS, SE, SQ, SOL, WASO, TST
Jernelov et al, 2012 [59]	Sweden	Adults with insomnia	133	47.9	6 w	Phone + bibliotherapy	RCT	ISI, DBAS, SE, SQ, SOL, WASO, TST, BTS, SRBQ
Koffel et al, 2016 [62]	United States	Veterans	18	48.5	5 w	Apps: “CBT-I Coach”	RCT	ISI
Lichstein et al, 2013 [15]	United States	Rural area adults with insomnia	5	65.8	5 w	Apps: “Skype” + CBT	Pre-post	ISI, HRDS, NWAK, SOL, SQ, WASO
Kauffman 2016 [61]	United States	Menopausal status with insomnia	106	54.85	24 w	Phone + CBT-I	RCT	ISI, PSQI, SE, SOL, TST, WASO
Mendelson et al, 2014 [64]	France	OSA patients	107	63	4 w	Researcher-built app + CPAP	RCT	ESS
Stremler et al, 2006 [67]	Canada	First-time mothers	30	31.85	6 w	Phone + sleep education	RCT	GSDS
van Dron-gelen et al, 2014 [22]	Netherlands	Airline pilots	502	40.9	6 m	App: “More energy”	RCT	PSQI
Vuletic el al., 2016 [70]	United States	Soldiers With MTBI	356	29.35	6 m	Telephone-based problem-solving treatment	RCT	PSQI, SE, SOL, SQ

^a^CUD: cannabis use disorders; MTBI: mild traumatic brain injury; OSA: obstructive sleep apnea.

^b^CBT-I: Cognitive Behavioral Therapy for Insomnia; CPAP: continuous positive airway pressure.

^c^RCT: randomized controlled trial.

^d^AHI: Apnea-Hypopnea Index; BTS: bed time stress; DBAS: Dysfunctional Beliefs and Attitudes about Sleep scale; ESS: Epworth Sleepiness Scale; GSDS: General Sleep Disturbance Scale; HRSD: Hamilton Rating Scale for Depression with sleep; ISI: Insomnia Severity Index; PSQI: Pittsburgh Sleep Quality Index; NWAK: number of awakenings; SE: sleep efficiency; SOL: sleep-onset latency; SQ: sleep quality; SQ_PSQI: extracted sleep quality score based on PSQI; SRBQ: Sleep-Related Behavior Questionnaire; SSR: Sleep Satisfaction Rate; TST: total sleep time; WASO: wakefulness after sleep onset.

**Table 2:** Summary of sleep measurement tools and study design for mobile phone sleep intervention included in the systematic review (N=16).

Measurement	Scales	Case study & pre-post test (n=4)	RCT (n=12)		
			Standard treatment: CBT-I & CPAP (n=5)^a^	Other recognized treatment (n=7)	Waitlist (n=4)^b^
Apnea-Hypopnea Index (AHI)	Score (total apneas event/TST)	0	2 [38,54]	0	0
Bed time stress (BTS)	Scored on scale 0-5	0	0	1 [59]	1 [ 59]
Dysfunctional Beliefs and Attitudes about Sleep Scale (DBAS)	30 items with scale 1-10 (total: 300)	0	1 [57]	1 [59]	2 [57,59]
Epworth Sleepiness Scale (ESS)	8 items with scale 0-3 (total: 24)	1 [41]	2 [38,54]	1 [64]	0
General Sleep Disturbance Scale (GSDS)	21 items with scale 0-7 (total:147)	0	0	1 [67]	0
Hamilton Rating Scale for Depression with sleep (HRSD)	Total 21 items:^c^: 10 items with scale 0-4, 2 items with scale 0-3, and 10 items with scale 0-2 (total: 66)	1 [15]	0	0	0
Insomnia Severity Index (ISI)	7 items with scale 0-4 (total: 28)	1 [15]	3 [15,57,61]	2 [59,62]	2 [57,59]
Number of awakenings (NWAK)	Frequency of awakening	1 [15]	0	0	0
Pittsburgh Sleep Quality Index (PSQI)	7 components calculated from 9 questions with scale 0-3 (total: 21)	1 [13]	2 [57,61]	2 [55,70]	3 [22,55,57]
Sleep efficiency (SE)^c^	Percentage (TST/total time in bed)	0	2 [57,61]	2 [59,70]	2 [57,59]
Sleep-onset latency (SOL)	Minutes	1 [15]	2 [57,61]	2 [59,70]	2 [57,59]
Sleep quality (SQ)^c^	Scored on scale 1-5	1 [15]	1 [57]	2 [59,70]	2 [57,59]
Extracted sleep quality score based on PSQI (SQ_PSQI)	8 items with scale 1-4 (total: 32)	0	0	1 [21]	1 [21]
Sleep-Related Behavior Questionnaire (SRBQ)	32 items with scale 1-5 (total: 160)	0	0	1 [59]	1 [59]
Sleep Satisfaction Rate (SSR)^c^	Scored on scale 0-3	1 [46]	0	0	0
Total sleep time (TST)	Hours	0	2 [57,61]	2 [21,59]	3 [21,57,59]
Wakefulness after initial sleep onset (WASO)	Minutes	1 [15]	2 [57,61]	1 [59]	2 [57,59]

^a^CBT-I: Cognitive Behavioral Therapy for Insomnia; CPAP: continuous positive airway pressure.

^b^Three articles have two comparison groups (waitlist vs other).

^c^Higher scores indicate lesser severity.

Table 2 shows the number of studies based on study design and sleep measurement tools. Several questionnaires were used to evaluate various aspects of sleep quality and quantity.

Specifically, the Pittsburgh Sleep Quality Index (PSQI) [13,21,22,55,57,61,70] had a list of items to evaluate sleep quality and it was the most frequently used measure in our review. In addition, three studies used a sleep quality [15,57,59] measure that consisted of a simple question about the participant’s perception about sleep quality using a five-point scale. Moreover, five studies [15,21,57,59,61] used measures of sleep quantity: (1) sleep-onset latency (n=4; the length of time to transition from full wakefulness to sleep completely) [15,57,59,61], (2) number of awakenings (n=1, the frequency of awakening during sleep) [15], (3) wake after sleep onset (n=4; the amount of time [eg, minutes] one wakes up during sleep) [15,57,59,61], and (4) total sleep time (n=4; total length of sleep time) [21,57,59,61]. Total sleep time is also used to calculate sleep efficiency [57,59,61]; that is, the percentage of total sleep time per total bed time. For the measurement of behavioral aspects of sleep, some researchers used the Epworth Sleepiness Scale (ESS) (n=4) [38,41,54,64] to capture participant sleepiness. Also two additional studies measured other specific sleep-related behaviors, such as the Sleep-Related Behavior Questionnaire (SRBQ) [59], the Dysfunctional Beliefs and Attitudes about Sleep Scale (DBAS) [57,59], and Sleep Satisfaction Rate (SSR) [46]. Finally, some sleep measurement tools focused on specific sleep disorders such as the severity of insomnia. The Apnea-Hypopnea Index (AHI) [38,54] is used to evaluate sleep apnea, and the General Sleep Disturbance Scale (GSDS) [67] is meant to examine factors that interrupt sleep. Two studies [15,59] used tools to examine the emotional aspects of sleep, such as bed time stress (BTS) levels [59], and the Hamilton Rating Scale for Depression with sleep (HRSD) [15]. All sleep outcome measurements were conducted by subjective measurement (ie, self-report questionnaire).

**Table 3:** Summary statistics: quality assessment of each journal and the effects of intervention through *t* test by intervention type and sleep outcome measurement.

Author(s), year	Quality score	Study design: intervention type^a^	Sleep measurement^b^	Effect size, mean (SD)	95% CI	*P*
Anttalainen el al, 2014 [38]	7	RCT: mobile + CPAP vs standard CPAP	AHI	–1.9 (2.9)	–2.4, –1.4	<.001
			ESS	0.0 (1.7)	–0.3, 0.3	>.99
Babson et al, 2015 [13]	3	Pre-post	PSQI	–1.5 (2.4)	–6.2, 3.2	NA
Bauer et al, 2012 [41]	5	Pre-post	ESS	–1.7 (1.3)	–2.4, –0.9	.001
Chen et al, 2015 [46]	2	Case study	SSR			NA
Filion et al, 2015 [21]	8	RCT: smoking prevention text vs sleep or activity	SQ_PSQI	–0.2 (1.7)	–0.5, 0.1	.14
			TST (weekday)	0.6 (0.5)	0.5, 0.6	<.001
		Promotion text	TST (weekend)	0.6 (0.7)	0.5, 0.7	<.001
Fox el al, 2012 [54]	8	RCT: mobile + CPAP vs standard CPAP	AHI	–1.9 (4.3)	–2.9, –0.9	<.001
			ESS	–0.9 (5.1)	–2.1, 0.3	.13
Freeman et al, 2015 [55]	9	RCT: mobile vs waitlist	PSQI	–2.6 (1.5)	–2.9, –2.2	<.001
		RCT: mobile vs standard treatment	PSQI	–0.7 (1.4)	–1.0, –0.4	<.001
Ho et al, 2014 [57]	8	RCT: mobile + CBT-I vs waitlist	DBAS	–23.5 (15.1)	–25.5, –21.5	<.001
			ISI	–3.1 (1.8)	–3.3, –2.9	<.001
			PSQI	–2.3 (1.5)	–2.5, –2.1	<.001
			SE	4.5 (4.8)	3.8, 5.2	<.001
			SOL	–7.4 (12.9)	–9.2, –5.6	<.001
			SQ	–0.2 (0.2)	–0.2, –0.2	<.001
			TST	0.0 (8.8)	–1.2, 1.2	.99
			WASO	–10.8 (14.8)	–12.8, –8.8	<.001
		RCT: mobile + CBT-I vs standard CBT-I	DBAS	–0.3 (16.0)	–2.5, 1.9	<.001
			ISI	–1.1 (1.8)	–1.3, –0.9	<.001
			PSQI	–0.8 (1.5)	–1.0, –0.6	<.001
			SE	1.2 (4.8)	0.5, 1.9	<.001
			SOL	–7.1 (12.9)	–8.9, –5.3	<.001
			SQ	–0.1 (0.2)	–0.1, –0.1	<.001
			TST	–0.1 (8.8)	–1.3, 1.1	.74
			WASO	–7.7 (14.7)	–9.7, –5.7	<.001
Jernelov et al, 2012 [59]	9	RCT: mobile + bibliotherapy vs waitlist	BTS	–0.9 (0.4)	–1.0, –0.8	<.001
			DBAS	–56.4 (9.8)	–58.4, –54.4	<.001
			ISI	–8.8 (1.6)	–9.1, –8.5	<.001
			SE	15.3 (7.1)	13.8, 16.8	<.001
			SOL	–30.7 (20.4)	–35.0, –26.4	<.001
			SQ	0.9 (0.2)	0.9, 0.9	<.001
			SRBQ	–21.9 (4.8)	–22.9, –20.9	<.001
			TST	0.4 (0.5)	0.3, 0.5	<.001
			WASO	–30.1 (18.9)	–34.1, –26.1	<.001
		RCT: mobile+ bibliotherapy vs bibliotherapy only	BTS	–0.9 (0.4)	–1.0, –0.8	<.001
			DBAS	–37.0 (11.0)	–39.3, –34.7	<.001
			ISI	–4.5 (1.5)	–4.8, –4.2	<.001
			SE	10.2 (7.0)	8.7, 11.7	<.001
			SOL	–14.8 (21.8)	–19.3, –10.3	<.001
			SQ	0.5 (0.2)	0.5, 0.5	<.001
			SRBQ	–15.3 (5.4)	–16.4, –14.2	<.001
			TST	0.0 (0.5)	–0.1, 0.1	.69
			WASO	–21.1 (19.4)	–25.1, –17.1	<.001
Koffel et al, 2016 [62]	8	RCT: app use vs non app	ISI	3.4 (4.6)	1.2, 5.5	.008
Lichstein et al, 2013 [15]	6	Pre-post	HRSD	–7.0 (3.6)	–10.2, –3.8	.01
			ISI	–11.3 (3.3)	–14.2, –8.3	.002
			NWAK	–1.7 (2.1)	–3.5, 0.1	.14
			SOL	–18.4 (20.9)	–36.7, –0.1	.12
			SQ	0.5 (0.1)	0.4, 0.6	.001
			WASO	–23.8 (9.2)	–31.9, –15.7	.004
Kauffman 2016 [61]	8	RCT: mobile + CBT-I vs menopause education	ISI	–4.0 (5.6)	–5.1, –2.9	<.001
			PSQI	–1.6 (3.0)	–2.2, –1.0	<.001
			SE	3.2 (12.3)	0.8, 5.6	.009
			SOL	–11.9 (36.3)	–18.8, –5.0	.001
			TST	0.2 (1.2)	0.0, 0.4	.08
			WASO	–6.8 (47.3)	–15.8, 2.2	.14
Mendelson et al, 2014 [64]	8	RCT: app + CPAP vs standard CPAP	ESS	–0.2 (1.5)	–0.5, 0.1	.17
Stremler et al, 2006 [67]	8	RCT: mobile + education vs education	GSDS	–13.3 (8.2)	–16.2, –10.4	<.001
van Drongelen et al, 2014 [22]	9	RCT: app use vs non app	PSQI	–0.6 (1.3)	–0.7, –0.4	<.001
Vuletic el al, 2016 [70]	8	RCT: mobile + education vs education	PSQI	–1.5 (1.5)	–1.7, –1.4	<.001

^a^CBT-I: cognitive behavioral therapy for insomnia; CPAP: continuous positive airway pressure.

^b^AHI: Apnea-Hypopnea Index; BTS: bed time stress; DBAS: Dysfunctional Beliefs and Attitudes about Sleep Scale; ESS: Epworth Sleepiness Scale; GSDS: General Sleep Disturbance Scale; HRSD: Hamilton Rating Scale for Depression with sleep; ISI: Insomnia Severity Index; PSQI: Pittsburgh Sleep Quality Index; NWAK: number of awakenings; SE: sleep efficiency; SOL: sleep-onset latency; SQ: sleep quality; SQ_PSQI: extracted sleep quality score based on PSQI; SRBQ: Sleep-Related Behavior Questionnaire; SSR: Sleep Satisfaction Rate; TST: total sleep time; WASO: wakefulness after initial sleep onset.

**Figure 2:**
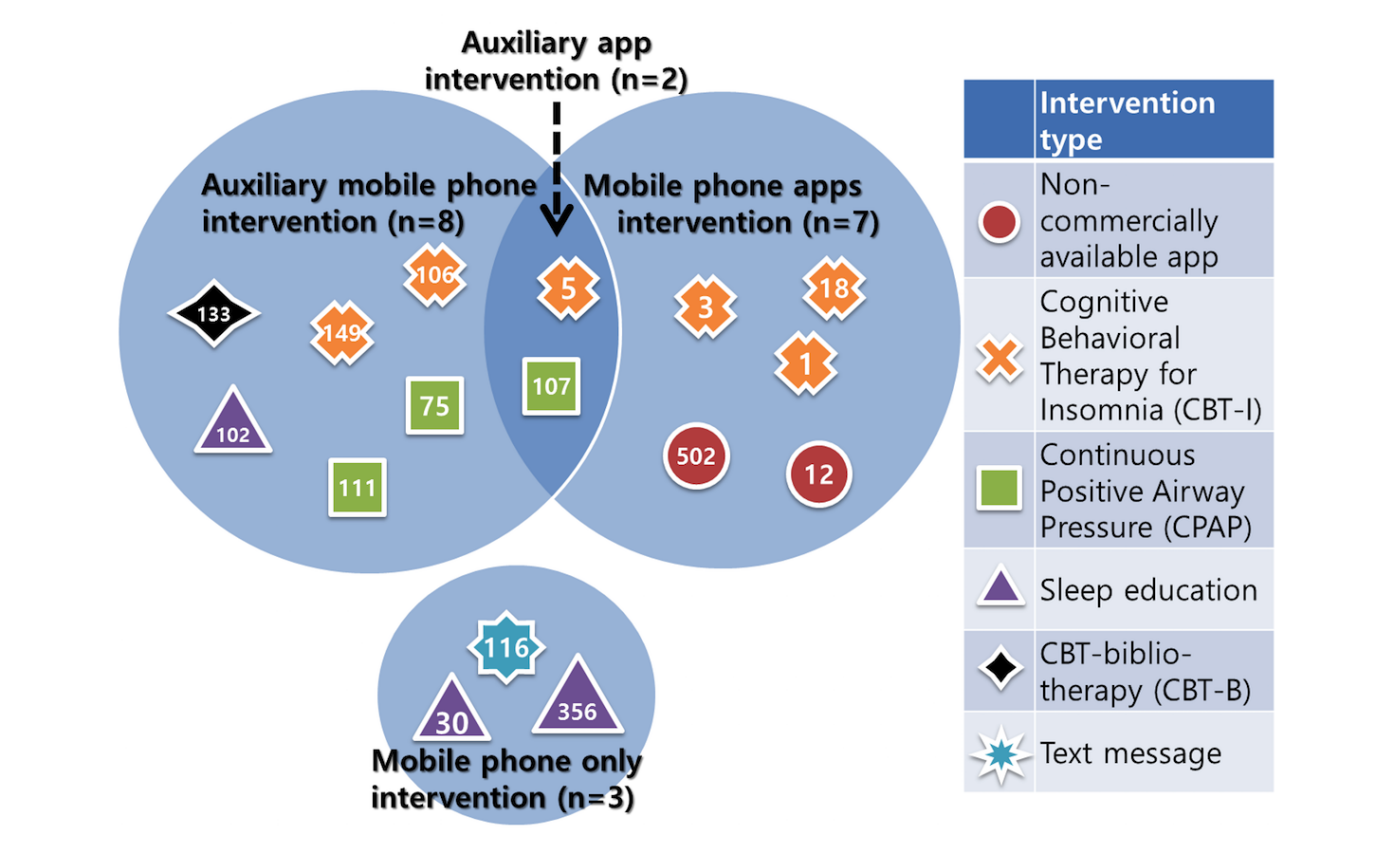
Sample size based on the intervention methods (N=16).

Table 3 provides a summary of the effectiveness of each sleep intervention. It is categorized by sleep outcome measurement, and the statistics can be comparable if there are more than two sleep measures. Specifically, half of studies [13,22,41,46,62,64,67,70] used a single sleep outcome measurement, while others had at least two or more measurements. Of nine studies [13,15,38,46,54,57,61,62,64] of standard treatment, five studies compared a mobile phone intervention and standard treatment intervention: (1) continuous positive airway pressure (CPAP) [38,54,64] and (2) cognitive behavioral therapy for insomnia (CBT-I) [57,61]. Five studies [13,22,41,46,61] focused on a mobile phone app. Two of these studies [13,61] used a “CBT-I coach” app that was developed based on CBT-I, which is considered one of the traditional standard treatments for insomnia (Figure 2).

**Table 4:** Quality assessment of studies to determine the impact of sleep intervention with mobile technology on sleep disorders.

Criteria item	Score, mean (SD)
1. Research question and objective were stated clearly	0.94 (0.24)
2. Definition of telehealth and/or mHealth was stated	0.18 (0.39)
3. A control group was included	0.82 (0.39)
4. Participants were randomly recruited from well-defined population	0.82 (0.39)
5. Sample size was >30	0.76 (0.44)
6. Attrition was analyzed and determined not to significantly differ by respondents’ baseline characteristics between control and experiment groups (<20%)	0.47 (0.51)
7. Baseline characteristics between control and intervention groups were similar	0.76 (0.44)
8. The intervention period was at least 4 weeks	0.94 (0.24)
9. The sleep disorder measurement tools were shown to be reliable and valid in previously published studies	0.88 (0.33)
10. Demographic information is available to control potential confounders for future analysis	0.82 (0.39)
Total study quality score^a^	7.41 (0.44)

^a^By summing up items 1 to 10 (range 3-10).

### Study Quality

Table 4 summarizes the results of the quality assessment of the studies included in this review. The quality score of the final articles ranged from 3 to 10 out of a possible score of 10, with an overall mean score of 7.41 (SD 0.44). The distribution of quality scores differed substantially across each criterion. Of 16 articles, 12 articles were considered high quality [21,22,38,54,55,57,59,61,62,64,67,70], two articles moderate quality [15,41], and two articles poor quality [13,46]. Most of the studies had clear research questions and objectives [13,15,21,22,38,41,46,54,55,57,59,62,64,67,70], and used valid sleep measurement tools [13,15,21,22,38,41,54,55,57,59,61, 62,64,70] for outcome measures after 4 weeks [15,21,22,38, 41,46,54,55,57,59,61,62,64,67,70]. All RCTs [21,22,38,54,55, 57,59,61,62,64,67,70] satisfied items 3 (ie, existence of control group) and 5 (ie, sample size greater than 30). The mean intraclass correlation (ICC) between peer reviewers for quality assessment was ICC 0.987 (95% CI 0.964-0.996, *P*<.001).

### Effectiveness of Mobile Phones Usage as a Sleep Intervention Tool

The mobile phone can be used as a tool to effectively deliver and enhance traditional behavioral interventions due to its portability. We found eight articles that described the advantages of using mobile phones to improve traditional intervention methods for sleep disorders [38,54,55,57,59,61,67,70].

#### Mobile Phone Usage as Alternative Intervention Tools

Mobile phones are a tool for intervention studies due to the portability for the participant. Within the results of our review, two studies focused on the effectiveness of mobile phones as intervention tools.

Stremler et al [67] was the first published research article that used a mobile phone as a behavioral-educational strategy tool for a sleep intervention. The focus of the study was to evaluate the feasibility, acceptability, and effects of a mobile phone intervention on sleep in the early postpartum period. During the 6-week intervention period, the intervention group was provided a well-established booklet, in-depth counseling, and a nurse to call for sleep advice, whereas the control group was only provided a one-page pamphlet and brief meeting session. Because the primary target subjects were first-time mothers and their children, researchers used GSDS to evaluate the sleep disturbances in employed women. As a result, the sleep intervention group had lower GSDS scores as much as 13.3 points compared to the control group (*t*_28_=–8.84, *P*<.001).

Similarly, Vuletic et al [70] had two intervention groups: (1) a group with 12 biweekly “telecounseling” and (2) an “education-only” group with an educational brochure. The research team developed the telephone-based problem-solving treatment, which was a sleep intervention that related to sleep quality to improve post-deployment soldier’s traumatic brain injury. Since the researchers used various sleep outcome measurements such as SOL, total sleep time, sleep disturbance, and daytime dysfunction that were part of PSQI, only PSQI was included for data analysis. After 6 months of intervention, the intervention group with telecounseling had significantly lower scores than the education-only intervention group (effect size=–1.54, SE 0.19; *t*_274_=–16.62, *P*<.001).

#### Mobile Phone Used as Auxiliary Equipment

Both CPAP and CBT-I are considered standard treatments for obstructive sleep apnea (OSA) [38,54] and sleep disturbances [13,57,62]. Three studies [38,54,61] applied mobile phones as supplementary tools in addition to these standard treatments.

Fox and colleagues [54] conducted a RCT to determine the effectiveness and adherence of telemonitoring and CPAP combined. To demonstrate the effectiveness of the intervention, AHI and ESS were used to measure sleep outcome. Researchers found that participants in the intervention group had an improvement in adherence to CPAP, but no significant differences were found for AHI and ESS between the control and intervention groups. Based on *t* test results, in addition, the effect size of AHI showed significantly decreased (*t*_73_=–3.82, *P*<.001).

Anttalainen et al [38] had a similar research method as Fox and colleagues’ [54], such as intervention duration, study design, intervention methods, presence of a control group, and sleep outcome measurement. Anttalainen et al did not find a statistically significant difference in ESS between groups, whereas participants in the intervention group had a significantly higher score on the AHI than standard in-person treatment (*t*_109_=–6.91, *P*<.001). Because ESS between standard CPAP and mobile-supported CPAP were not significantly different, the effects of mobile-supported CPAP did not differ from standard CPAP.

Kauffman [61] conducted a RCT to determine the effectiveness of telephone-based CBT-I to determine the effectiveness of telephone-based CBT-I compared to menopause education control. The target population was menopausal middle-aged women with moderate insomnia (ISI>15). Based on the *t* test results, there was a significantly different effect size for the index type sleep measurements between the telephone-based CBT-I and menopause education control (ISI: *t*_104_=–7.33, *P*<.001; PSQI: *t*_104_=–5.54, *P*=.007), whereas sleep time measures were not significant such as sleep onset latency, wake after sleep onset, total sleep time, and sleep efficiency.

#### Multidimensional Approach to Mobile Phone Usage

Three studies [55,57,59] examined the effectiveness of mobile phone usage as an alternative intervention tool and as an auxiliary equipment.

Jernelov et al [59] evaluated the effectiveness of mobile phone support with CBT bibliotherapy (CBT). To examine the effectiveness of mobile phone support, a total of 133 participants were randomly allocated to three comparable groups (44 for CBT bibliotherapy with mobile support; 45 for only CBT bibliotherapy; 44 for the control group). After the 6-week intervention, a total of 116 participants completed the follow-up assessment (40 for CBT bibliotherapy with mobile support; 37 for CBT bibliotherapy; 39 for the control group), with an attrition rate of 12.78%. Participants in the mobile-supported CBT bibliotherapy group had significant improvements in all sleep measurements compared to the control group (BTS: *t*_86_=–20.80, *P*<.001; DBAS: *t*_86_=–53.96, *P*<.001; ISI: *t*_86_=–50.64, *P*<.001; SE: *t*_85_=20.18, *P*<.001; SOL: *t*_85_=–14.06, *P*<.001; SQ: *t*_85_=39.59, *P*<.001; SRBQ: *t*_86_=–42.81, *P*<.001; TST: *t*_85_=6.77, *P*<.001; WASO: *t*_85_=–14.83, *P*<.001), and even CBT bibliotherapy only (BTS: *t*_87_=–21.95, *P*<.001; DBAS: *t*_87_=–31.74, *P*<.001; ISI: *t*_87_=–28.88, *P*<.001; SE: *t*_87_=13.78, *P*<.001; SOL: *t*_87_=–6.39, *P*<.001; SQ: *t*_87_=24.41, *P*<.001; SRBQ: *t*_87_=–26.77, *P*<.001; WASO: *t*_87_=–10.26, *P*<.001), except TST (*t*_87_=0.685, *P*=.84).

Although Ho et al [57] had a similar study design and approach to Jernelov and colleagues [59], Ho et al had a different intervention tool: the comparison between the self-help CBT-I with the mobile phone support and self-help CBT-I only. Of importance was that this study had a relatively higher attrition rate (49%) because this study was community based. To be specific, although the baseline participants were high (N=302; 103 for CBT-I with mobile support; 104 for CBT-I; 105 for control group), the total number of participants that completed the follow-up assessment was similar with other RCTs (n=149; 44 for CBT-I with mobile support; 39 for CBT-I; 66 for control group). The intervention had two follow-up sessions (at 4 weeks and at 12 weeks), but the waitlist group did not have 12-week evaluation. Therefore, the statistics in Table 3 were based on the follow-up session at 4 weeks to compare whether both interventions significantly improved the sleep measurement score compared to the initial assessment. From the *t* test, several sleep outcome measures indicated that mobile phone-supported intervention had a statistically significant improvement compared to the waitlist control group (PSQI: *t*_206_=–21.93, *P*<.001; ISI: *t*_206_=–24.37, *P*<.001; DBAS: *t*_206_=–22.48, *P*<.001; SOL: *t*_206_=–8.27, *P*<.001; WASO: *t*_206_=–10.56, *P*<.001; SE: *t*_206_=13.57, *P*<.001; and SQ: *t*_206_=–14.14, *P*<.001) and also the standard treatment intervention (PSQI: *t*_205_=–7.63, *P*<.001; ISI: *t*_205_=–8.64, *P*<.001; SOL: *t*_205_=–7.94, *P*<.001; WASO: *t*_205_=–7.53, *P*<.001; SE: *t*_205_=3.62, *P*<.001; and SQ: *t*_205_=–7.85, *P*<.001).

Freedman et al [55] established the “Envision the Rhythms of Life” program, which is a group intervention to increase the quality of life for breast cancer survivors, and also to evaluate its advantages on aspects of quality of life. A total of 118 participants were randomly assigned to three groups (48 participants for the in-person delivery; 23 participants for the mobile phone-supported teleconferencing; 47 participants for waitlists) at the beginning of the study, and 102 participants remained (40 participants for the in-person delivery; 19 participants for the mobile phone-supported teleconferencing; 43 participants for the waitlists) at the end of the study. According to the *t* test, participants in the mobile phone-supported group had a significant increase in PSQI scores among breast cancer survivors compared to both control groups (standard treatment: mean –0.68, SD 1.39; *t*_73_=–4.11, *P*<.001; waitlist: mean –2.55, SD 1.53; *t*_73_=–13.97, *P*<.001). In addition, authors performed the linear multilevel modeling analysis, and found that PSQI was only considerable for group effect (*P*<.001), not for time (*P*=.35) or group×time (*P*=.30).

#### Text Message for Sleep Intervention

Filion et al [21] used a text message-based intervention for young adult smokers to promote better sleep and physical activity habits. A total of 164 baseline participants were assigned to two groups: (1) sleep/physical activity group (n=63) and (2) smoking cessation group (n=101). A total of 129 participants completed the study (smoking cessation group: n=77; sleep group: n=44). Participants in the sleep/physical activity group received sleep- and activity-related messages for 6 weeks, whereas the smoking cessation group received quitting smoking-related messages. Although the difference of the PSQI score between the smoking group (mean –1.47, SD 1.77) and sleep group (mean –1.7, SD 1.48) was not significant (*P*=.60), the amount of sleep was significantly different (*P*=.03). Specifically, the amount of sleep (hours) for the sleep/physical activity group increased (mean 0.52, SD 0.44), whereas sleep among the smoking cessation group decreased (mean –0.03, SD 0.46).

#### Effectiveness of Mobile Phone Apps as a Sleep Intervention Tool

As a measurement tool for sleep disorder intervention, mobile phone apps are able to perform diverse functions (ie, tracking [13,41,46,62], sleep advice for behavioral change [13,15,41,46], and optimized alarms [13,22,62]). In our review, seven studies evaluated the effectiveness of these app-based interventions [13,15,22,41,46,62,64].

##### Mobile Phone Apps as Auxiliary Equipment

Lichstein et al [15] used the Skype app for teleconferencing in addition to CBT as a sleep intervention. The purpose of the study was to examine the feasibility and effectiveness of interventions to address challenges faced by rural older adults with comorbid conditions, namely insomnia and depression. The researchers demonstrated the limitation of mobile phone apps for older population (age: mean 65.8, SD 10.4 years). From the *t* test, participants in the Skype group had significant improved sleep quality (sleep quality: *t*_4_=9.7, *P*<.001), less insomnia (ISI: *t*_4_=–7.54, *P*=.002), less wakefulness after sleep onset (*t*_4_=–5.77, *P*=.004), and lower HRSD scores (*t*_4_=–4.33, *P*=.01). Despite the improvement in sleep quality, this study had a higher attrition rate; only five of 18 baseline participants completed the entire study (Multimedia Appendix 2).

Mendelson et al [64] evaluated whether a combination of the mobile phone app using telemedicine and CPAP had better performance compared to standard care (CPAP only). After 4 weeks of intervention, a total 82 of 107 baseline participants (76.6%) completed the entire test procedure, and 54 participants adhered to CPAP for more than 4 hours. Although the ESS was statistically significantly different between pretest and posttest (combined intervention: mean –2.3, SD 4.0, *P*<.05; standard care: mean –2.1, SD 4.1, *P*<.001), the effectiveness of treatment did not differ in each intervention.

##### Mobile Phone Apps Developed by Researchers to Improve Sleep Behavior

Bauer et al [41] were the first to assess the use of mobile phone apps for sleep intervention programs. The research team developed the “Shuteye” app in 2011, which offered real-time recommendations to promote awareness related to healthy sleep behaviors. They also highlighted the following as being important for effective sleep apps: design of the app, adherence of mobile phone, appearance, awareness of app usage, and learnability (ie, user-friendly interface). For the intervention study, ESS was measured for 12 participants before and after the intervention. We calculated the changes in participant scores pre- and postintervention based on the raw data provided by the research team. Based on the data in the articles, we performed the paired *t* test, and ESS decreased after intervention (mean –1.67, SD 1.32; *t*_11_=–4.36, *P*=.001), although this was not found to be statistically significant, most likely due to the small sample size.

Van Drongelen et al [22] conducted a RCT addressing sleep using a mobile phone app with international aircraft pilots. The “More Energy” app was invented to reduce pilots’ fatigue and also to improve their healthy behavior and sleep. During the 6 months of intervention, a total of 390 participants (77.7% of 502 baseline participants: 191 in the intervention group, 199 in waitlists) completed the study. The intervention program showed statistically significant fatigue improvement, and also showed a positive effect on sleep. We calculated the difference between the intervention group (score decreased by mean 0.2, SD 1.2) and waitlists (score increased by mean 0.37, SD 1.32); the PSQI score significantly improved (beta=–0.59, *P*=.001).

##### Mobile Phone Apps With CBT-I

Three studies [13,46,62] used mobile phone apps to reform CBT-I, which is one of the traditional standard treatments for improving sleep behavior.

Babson et al [13] used a mobile phone app version of CBT-I to determine the feasibility and efficacy for veterans using cannabis to treat sleep disorders. A total of four participants were included in the study (two in the intervention group; two in the control group). According to the *t* tests result, the mobile phone intervention did not significantly affect the PSQI score (*t*_1_=–0.87, *P*=.48). However, this study was considered a pre-post study because all participants in the control group dropped out during the test procedure. In addition to the loss of a follow-up evaluation in the control group, several limitations with the study, such as small sample size and the rarity of the target population (ie, veterans using cannabis treatment for sleep disorders), caused the authors’ skeptical conclusion of the ineffectiveness of mHealth intervention.

Chen et al [46] used the “Win-Win a Sleep” app, which was developed from CBT-I, and revealed effectiveness and feasibility of sleep interventions. In this case study, a participant who was female aged 63 years suffered from insomnia and used the app during a 5-week test period. Because the participant took hypnotic medications, researchers also considered the impact of decreasing the amount of drugs used to sleep treat disorders. The participant had better sleep satisfaction, from no preference (mean 1.5, SD 0.7) to good (mean 2.0, SD 0.0), based on the result of a Likert scale change (score range between 0=‘‘very bad’’ and 3=‘‘very good’’).

Koffel et al [62] recruited from the general population to conduct a RCT to determine the feasibility and acceptability of CBT-I coach apps. A total of 18 participants evaluated their ISI scores at each therapy session (five times), and also reported the adherence rate. We did not perform a *t* test because there was no distinguishable statistic of ISI included in the article. However, ISI scores tended to decrease based on figures that were analyzed by hierarchical linear models. There was a significant difference (*t*_15_=3.04, *P*=.008) in ISI scores between baseline and after the third session, even though the standard CBT-I group had lower ISI scores than the app users group.

##### Lack of Data for Performing Meta-Analysis

Although meta-analysis is a great way to examine the effectiveness of interventions with a specific parameter, we did not have enough articles to conduct a meta-analysis. Sleep measurements were not included in all articles selected for the systematic literature review. Because more than four articles were required to perform a meta-analysis, according to Table 2, there were not enough articles when stratified by sleep measurement and study design. Also, a sample size of N=2 or N=3 is insufficient to perform the publication bias test (Begg rank correlation test or Egger regression test) and to determine the level of heterogeneity.

## Discussion

### Summary of Evidence

The purpose of our systematic review was to investigate whether mobile phones are a feasible and usable tool to improve sleep disorders and sleep quality in intervention studies. We also assumed that auxiliary mobile phone use helps to enhance the performance of existing intervention methods.

This review presented the articles based on two intervention methods. The first method was utilizing mobile phone apps and the second was conventional mobile phone support through telephone calls or text messages. Although many mobile phone apps were used independently, the conventional mobile phone supporting methods were (1) to use the mobile phone itself as only an intervention tool (eg teleconferencing or telecounseling), or (2) to combine with another treatment (eg, CBT-I, CBT-B, and CPAP) to enhance the effectiveness of sleep interventions. In our review, six studies were related to CBT-I [13,15,46,57,61,62] and three studies to CPAP [38,54,64]. This indicated that sleep intervention studies using mobile phones have been developed based on the standard treatment, which has already shown reliability [33,53,71-73].

Among the sleep outcome measurements, PSQI was the most frequently reported, and PSQI scores of all seven studies [13,21,22,55,57,61,70] were significantly decreased in the intervention group using mobile phones compared to standard treatment and a waitlist group. Participants in the intervention groups with mobile phones had a mean decrease in PSQI scores of 1.73 points compared to the waitlist group, and a mean decrease of 0.97 points compared to other treatment groups. Although the difference of PSQI was statistically significant, clinicians must consider the clinical significance of a one-point change in PSQI scores. According to the Buysse et al [74], a score of 5 of 21 is the cut-off point for poor sleep. Thus, the clinical significance of a one-point difference depends on the baseline score.

The ISI was the second most frequently used sleep outcome measurement tool, and all five articles using the measure [15,57,59,61,62] found a statistically significant decrease in ISI score in the mobile phone use intervention group compared to non-mobile phone use group. Participants in the intervention group with mobile phones had a mean decrease in ISI scores of 5.09 points compared to the waitlist groups [57,59,62], and a mean decrease of 3.2 points in ISI scores compared to other treatments [57,59,61]. Because a change of 7 points in the ISI is interpreted as a different level of insomnia, a 3- or 5-point decrease in ISI scores might be considered a critical improvement. Although ESS, WASO, SOL, and TST were included in at least four studies, there was at least more than one study that reported a lack of statistical significance of intervention effect [15,38,41,54,57,59,61]. Therefore, ISI and PSQI seem to be the most applicable measurement tools to investigate the effect of mobile phone intervention on sleep disorders.

In general, 87.5% (14/16) of the studies [15,21,22,38,41,54,55,57,59,61,62,64,67,70] reviewed supported the evidence of capability and efficacy of mobile phone usage interventions. When mobile phones were used as auxiliary equipment, the intervention that applied telephone calls or mobile phone apps clearly demonstrated equal or enhanced efficacy for sleep quality and index score compared to the traditional sleep intervention [15,38,54,55,57,59,61,64,67,70]. This difference was seen not only for the waitlist control group [54,55,57,59], but also for the telephone-supported intervention and traditional treatment [15,38,61,64,67,70]. The advantage of mobile phone intervention was also illustrated in apps using intervention [13,22,41,46,62] or text message interventions [21].

### Limitations of Review

There are several limitations of the studies included in this review. First, there is no standardized study design, especially for the test period, procedure, and sleep intervention tools. For example, there is a limitation to use all mobile phone apps to compare each study because researchers used their own personally developed or nonpublicly available apps. As such, we were unable to make comparisons between interventions due to differences in app functions and interfaces. Due to the heterogeneity of the study design and sleep measurement tools, it was difficult to compare the effectiveness of the sleep app interventions. This will be an issue for reproducibility in further research. Second, although it is a common limitation for RCT study designs, the uniqueness of each study’s target population limits further analysis and replication. For instance, results from studies with a cannabis disorder use group [13] and a post-deployment soldier with mild traumatic brain injury [70] were difficult to extrapolate to the general population. Third, there are few interventions using mobile technology that have been published in peer-reviewed journals, so we were limited with respect to the number of articles we could include in the study. Although many *t* test for each sleep measurement showed significant effectiveness of sleep interventions, the small number of studies for subgrouping limits performing a meta-analysis. Fourth, there is the possibility of missed articles. Because the mHealth market is growing rapidly, our search possibly missed some articles that used new mobile phone intervention tools. On the other hand, it also might be possible to miss some articles during the search stage due to the usage of different language or jargon unique to the evolving mHealth industry.

### Conclusion

This study has several strengths. To the best of our knowledge, this study was the first to review the effectiveness of mobile phone interventions on sleep quality, quantity, and sleep disorders. By focusing exclusively on the mobile phone itself, it allows us to tailor future mHealth interventions for sleep. Also, our study examined various aspects of sleep measurement tools that account for sleep quality, sleep quantity, and many sleep disorders such as insomnia and sleep latency. Additionally, our study provided evidence of the potential of mobile-based interventions for improving sleep disorders. Along with current research that support the benefits of cost-efficiency of mHealth interventions, these findings provide an impetus for further research examining the empirical evidence of sleep interventions using mobile phones.

In conclusion, our systematic review supports the evidence that mobile technology-based interventions are an effective tool to improve symptoms of sleep disorders and quality of sleep than traditional intervention without mobile phone. Also, we suggest the following research design for future sleep intervention studies: (1) PSQI and ISI as sleep outcome measurements, (2) RCTs, (3) compare with standard treatment (ie, CPAP, CBT-I), and (4) compare to a waitlist control group. In addition to intervention methods, because mobile phone apps vary and many of these apps are not being studied, it is important to perform a content analysis on commercially available apps to determine common functionalities prior to undertaking interventions [75]. Our finding was not only applicable to those with sleep disorders who need clinical care, but also to medical professionals who are interested in ways to determine effective sleep interventions. Moving forward, app developers and sleep experts need to develop evidence-based guidelines with behavioral change components for sleep apps to maximize their efficacy and to take advantage of mobile phones to apply to existing standard treatments for sleep interventions.

## Multimedia Appendix 1

Searching Keywords.

## Multimedia Appendix 2

Quality Assessment Score.

## Abbreviations

AHI: Apnea-Hypopnea Index
BTS: bed time stress
CINAHL: Cumulative Index to Nursing and Allied Health Literature
CPAP: continuous positive airway pressure
DBAS: Dysfunctional Beliefs and Attitudes about Sleep Scale
ESS: Epworth Sleepiness Scale
GSDS: General Sleep Disturbance Scale
HRSD: Hamilton Rating Scale for Depression with sleep
ISI: Insomnia Severity Index
OSA: obstructive sleep apnea
PRISMA: Preferred Reporting Items for Systematic Reviews and Meta-Analysis
PSQI: Pittsburgh Sleep Quality Index
RCT: randomized controlled trial
SE: sleep efficiency
SOL: sleep-onset latency
SQ: sleep quality
SRBQ: Sleep-Related Behavior Questionnaire
SSR: Sleep Satisfaction Rate
TST: total sleep time
WASO: wakefulness after initial sleep onset
