# Supplementary material for: Mobile Phone Interventions for Sleep Disorders and Sleep Quality: Systematic Review
Source: JMIR Mhealth Uhealth. 2017 Sep 7;5(9):e131. doi: 10.2196/mhealth.7244 (PMC5608984; doi:10.2196/mhealth.7244)
Supplement: Supplementary file 1 [file mhealth_v5i9e131_app1.pdf]

Sleep	1. sleep*<br>2. (apneas OR apnea)<br>3. (sleep talk*)<br>4. sleep disorder<br>5. insomnia<br>6. snore<br>7. parasomnias<br>8. (sleep walk*)<br>9. sleep quality<br>10. sleep latency
[Sleep] = (1 OR 2 OR 3 OR 4 OR 5 OR 6 OR 7 OR 8 OR 9 OR 10)	
mhealth/telemedicine	11. ((mobile or smartphone or phone) AND (Apps OR application))<br>12. (mhealth or mobile health)<br>13. (telehealth OR tele health)
[mhealth/telemedicine] = (11 OR 12 OR 13)	
Study design	14. (intervention*)<br>15. (randomised OR randomized OR randomly)<br>16. (pre test OR pretest OR pre intervention OR post intervention OR post test OR posttest)<br>17. (control* OR before stud* OR after stud* OR follow up assessment)<br>18. Cohort<br>19. comparative stud*<br>20. control group<br>21. (evaluat* OR treatment OR process OR program*)
[Study design] = (14 OR 15 OR 16 OR 17 OR 18 OR 19 OR 20 OR 21)	
Final search term: [Sleep] AND [Mhealth/telemedicine] AND [Study design]	
