# Supplementary material for: Mobile Phone Interventions for Sleep Disorders and Sleep Quality: Systematic Review
Source: JMIR Mhealth Uhealth. 2017 Sep 7;5(9):e131. doi: 10.2196/mhealth.7244 (PMC5608984; doi:10.2196/mhealth.7244)
Supplement: Supplementary file 2 [file mhealth_v5i9e131_app2.pdf]

	Quality assessment items										
	**#1**	**#2**	**#3**	**#4**	**#5**	**#6**	**#7**	**#8**	**#9**	**#10**	**Total**
Anttalainen el al., 2014	1	0	1	1	1	0	1	1	1	0	7
Babson et al., 2015	1	0	1	0	0	0	0	0	1	0	3
Bauer et al., 2012	1	0	0	1	0	1	0	1	1	0	5
Chen et al., 2015	1	0	0	0	0	0	0	1	0	1	3
Filion et al., 2015	1	0	1	1	1	0	1	1	1	1	8
Fox el al., 2012	1	0	1	1	1	0	1	1	1	1	8
Freeman et al., 2015	1	0	1	1	1	1	1	1	1	1	9
Ho et al., 2014	1	0	1	1	1	0	1	1	1	1	8
Jernelov et al., 2012	1	0	1	1	1	1	1	1	1	1	9
Koffel et al., 2016	0	0	1	1	1	1	1	1	1	1	8
Lichstein et al., 2013	1	0	1	0	1	1	1	1	1	1	8
McCurry et al., 2016	1	1	0	1	0	0	0	1	1	1	6
Mendelson et al., 2014	1	0	1	1	1	1	1	1	1	1	9
Stremler et al., 2006	1	0	1	1	1	1	1	1	0	1	8
van Dron-gelen et al., 2014	1	1	1	1	1	0	1	1	1	1	9
Vuletic el al., 2016	1	0	1	1	1	0	1	1	1	1	8
